# Re: Serum tissue factor as biomarker for renal clear cell carcinoma: a comment

**DOI:** 10.1590/S1677-5538.IBJU.2017.0530

**Published:** 2018

**Authors:** Beuy Joob, Viroj Wiwanit

**Affiliations:** 1 Sanitation 1 Medical Academic Center, Bangkok Thailand; 2 Hainan Medical University, China; 3 Faculty of Medicine, University of Nis, Serbia; 4 Joseph Ayo Babalola University, Nigeria

*To the editor,*

We read the publication on “Serum tissue factor (TF) as a biomarker for renal clear cell carcinoma”. Silva et al. noted that “TF appears to be a useful serum marker for the presence of clear cell RCC (1)”. Indeed, to conclude that TF is an important biomarker, there should be a complete evaluation on this diagnostic test. Sensitivity and specificity should be studied. Also, some confounding medical disorders such as cardiovascular problem or hemoglobin disorder can affect TF level (2, 3). The conclusion that the TF is a useful biomarker for renal clear cell carcinoma by Silva et al. might be too early. Nevertheless, if the diagnostic property of TF is acceptable, there is still the left question of its cost effectiveness.
